# American Dental Association

**Published:** 1892-05

**Authors:** 


### AMERICAN DENTAL ASSOCIATION.

*August 6, 1891.—Third Day.—Afternoon Session.*

The election of officers for the ensuing year took place, with the following result:

President, W. W. Walker; First Vice-President, J. D. Patterson; Second Vice-President, S. C. G. Watkins; Corresponding Secretary, Fred. A. Levy; Recording Secretary, Geo. H. Cushing; Treasurer, A. H. Fuller.

*Executive Committee.*—J. N. Crouse, G. W. McElhaney, V. H. Jackson.

Place of meeting next year, Niagara Falls.

*August 6, 1891.—Third Day.—Evening Session.*

Reading of the minutes by the secretary, which were approved.

Dr. Barrett read the report of the Committee on the President's

Address, and also the recommendations for changes in the constitution and by-laws.

Dr. Fillebrown offered the following, which was adopted:

"*Resolved*, That the Committee on the Revision of the Constitution be continued, and that any suggestions proposed by members, received by the committee within thirty days after the mailing of the report to members, may be incorporated in the final report, which will appear in the published *Transactions*, and such publication shall be deemed sufficient notice of proposed amendments, so that a vote may be taken upon them at the next annual meeting."

On motion of Dr. Taft, Drs. J. B. Rich and Corydon-Palmer, who have each been in practice for over fifty years, were made permanent members of the Association, without the payment of dues.

*Dr. Smith.*—Mention was made during the report of Section VI. that contributions would be called for to continue the work of examining prehistoric crania. The money has been exhausted, and the committee asks for an additional allowance of five hundred dollars. I would, therefore, make a motion to that effect.

This was adopted.

Dr. Ottofy moved that Section VI. be authorized to ask for contributions in money from the various State societies, to assist in the prosecution of this work.

Adopted.

Dr. Ottofy also moved that the transactions of the American Dental Association be sent to all societies that have already contributed, or will contribute, towards the prosecution of this investigation.

Adopted.

Dr. Taft, of the Committee on Necrology, reported on the death of Dr. William H. Atkinson. Drs. Fredericks, Horton, Barrett, Butler, Morgan, Crawford, Patterson, Stockton, Corydon-Palmer, and Dwinelle spoke in eulogistic terms of the deceased.

Report accepted.

Dr. Marshall moved that a committee of two be appointed to confer and co-operate with the other societies which are taking steps to perpetuate the memory of Dr. Atkinson.

Adopted.

SECTION III.—OPERATIVE DENTISTRY.

Dr. V. H. Jackson read a paper, entitled "Methods of Regulating Teeth," of which an abstract follows:

Since reading a paper before this Association some time since, in which was described the system of using removable spring appliances, a number of methods have been used, some of which I will mention in my paper this evening. Only a portion of the alveolar process is developed until the teeth are fully erupted, and for this reason a slight pressure will change their position. Several methods were adopted with partial satisfaction for correcting irregularities of the teeth in young children, but none have been as successful as the crib.

Impression of irregularities are usually more satisfactory when taken with some of the compounds, as the surface of the model is more smooth than when taken in plaster, and is more agreeable to the patient. If the teeth are long, and there is danger of the compound dragging while being removed, a quantity of pulverized soapstone should be sifted over the surface before taking the impression.

If the second or third molar is inclined forward into the space formerly occupied by the first molar, the difficulty can be overcome by placing a rounded piece of soft compound between it and the next tooth, and allowing it to harden in such shape that there will be no undercuts. When well shaped and sufficiently hard, the impressions should be covered with one or two layers of paper, and then removed and placed together. If the operation is well performed, a perfect impression is the result. A little water injected under the lip will relieve the tendency to suction, and thus remove the danger of drawing the compound up in the centre, which often spoils the impression.

Since first referring to the system of retaining plates and cribs for regulating teeth, published in the *Dental Cosmos*, it has been constantly employed with various improvements. The models are first prepared by carving the plaster well away from the necks of the teeth that are selected as anchors. A piece of any metal suited to the purpose is formed to either or both the lingual or labial surfaces of the teeth. Very thin gold or German silver may be used. After the metal is cut to the proper form, it is contoured, which is made an easy task by using the contouring-pliers made by the S. S. White Dental Manufacturing Company. If the adjoining teeth are to be used as anchors, the metals are arranged on it and united. A spring wire, about No. 20, is then formed, as described in the previous paper, in making the simple crib. The wire passes around the teeth, as the case may require, and is formed so it will fit the labial side of the tooth, with both ends passing over the arch, resting on the metal described, but should be made to fit loosely, so as not to injure the model when removing it. First bend the wire at right angles, like this [illustrating], leaving the width between the parallel sides equal to the width of the tooth to be clasped. The part that is to clasp the neck of the tooth is then curved, so that it will be perfectly adapted to the labial side of the teeth.

Dr. Jackson then described the method in detail, showing numerous illustrations and charts.

About three years since it was observed that when copper was used in small quantity, in appliances that were retained for a long time, there appeared to be no injury in the way of structural changes or decomposition of food particles. The continued use of copper has proven its detersive properties. While relating the advantage of using copper recently, Dr. Elliott, of Sag Harbor, exhibited a copper-plate that he was wearing, that has copper covering the portion of the rubber which would come in contact with the natural teeth. The teeth were extremely sensitive at one time, but he has suffered no inconvenience since using the copper.

The extraction of teeth that is so common, to prevent crowding, should be discarded. If there is insufficient room for the eruption of the teeth, it is best to expand the arch, which is easily accomplished by means of the crib. Correcting the position of irregular inferior incisors will often correct the position of the superior incisors, if done while they are erupting.

Dr. Jackson, of Ann Arbor, Michigan, recommends to the profession a spring wire crib attached to a plate passing over one or more teeth on either side of the arch, for retaining obturators, and thus avoiding injury to the tooth-structure. (See *Journal of Dental Science*, vol. xi. p. 134.)

The advantage of the crib system for young patients is that it is easily retained and causes no inconvenience. The model is not injured in making the appliance, and is preserved as a record for future measurements and study. The same method is also recommended in cases of fracture.

Dr. Jackson then gave a number of illustrations, and specified several cases of his own which had been successful, and expressed the belief that the general adoption of this method could be anticipated in the near future.

Dr. N. S. Hoff, as secretary of the section, read its regular report, of which an abstract also follows:

As even a brief synopsis of the work done would make the report very lengthy, only a brief *résumé* will be presented. It is a fact that the discussions which usually follow the reading of a paper often transform an apparently indifferent paper into one of great value.

Probably the most notable contribution has been the series of papers contributed to the *Dental Cosmos* on the subject of "Management of the Enamel Margins," by Dr. Black. Another interesting paper, entitled "The Preparation of Teeth for Filling," was read at the Illinois State Society meeting by Dr. Noyes, and elicited a long and profitable discussion.

Papers by Drs. Wilson and Dwinelle caused interesting discussions in the New York Odontological Society on the subject of gold fillings, contour-fillings, etc. Dr. C. M. Gingrich presented a paper at the Maryland State Dental Society on "Cohesive Fillings." Dr. Ames read a paper at the Mississippi Valley Dental Society on the "Use of Copper Amalgam." Dr. Miller gave the result of his research in regard to oxyphosphate cements in the *Dental Cosmos* of January, 1891, and stated there that he is pleased to find that the subject of immediate root-filling is fast becoming one of the lost arts.

In the *Dental Cosmos* there is also to be found an article on "The Destruction of the Dental Pulp, and Methods of Treatment of Teeth discolored by its Retention in the Pulp-Chamber or Canal," by Dr. A. W. Harlan.

Dr. Kirk, in the July, 1891, number of the *Dental Cosmos*, recommends a new remedy (aristol), which is more agreeable to use than iodoform in pyorrhœa alveolaris, alveolar abscess, and as a dressing for root-canals.

The following books have also been published during the year: "Micro-Organisms of the Human Mouth," by Dr. W. D. Miller; "Compendium of Practical Dentistry," by B. H. Catching, D.D.S.; "A Compend of Dental Pathology," by George W. Warren; "Treatise on the Irregularities of the Human Teeth, and their Correction," by Dr. J. N. Farrar, M.D., D.D.S.; "Descriptive Anatomy of the Human Teeth," by G. V. Black, M.D., D.D.S.; "Dental Surgery and Pathology," by H. Sewill, M.R.C.S.

Dr. Custer read a report on the subject of obtundents and local anæsthetics used to obtund sensitive dentine, and mentioned several agents which are being widely used, the degrees at which they volatilize, and the effectiveness of each.

Adjourned.

